# Encapsulation of Menthol and Luteolin Using Hydrocolloids as Wall Material to Formulate Instant Aromatic Beverages

**DOI:** 10.3390/foods12102080

**Published:** 2023-05-22

**Authors:** Laura Sofía Mora-Flórez, Daniel Cabrera-Rodríguez, María Hernández-Carrión

**Affiliations:** Grupo de Diseño de Productos y Procesos (GDPP), Department of Chemical and Food Engineering, Universidad de los Andes, Bogotá 111711, Colombia; ls.moraf@uniandes.edu.co (L.S.M.-F.); d.cabrera@uniandes.edu.co (D.C.-R.)

**Keywords:** encapsulation, Scanning Electron Microscopy, image analysis, controlled release, in vitro digestion, functional food, hydrocolloids

## Abstract

Aromatic plants represent about 0.7% of all medicinal plants. The most common are peppermint (main active ingredient: menthol) and chamomile (main active ingredient: luteolin), which are usually consumed in “tea bags” to make infusions or herbal teas. In this study, menthol and luteolin encapsulates using different hydrocolloids were obtained to replace the conventional preparation of these beverages. Encapsulation was carried out by feeding an infusion of peppermint and chamomile (83% aqueous phase = 75% water − 8% herbs in equal parts, and 17% dissolved solids = wall material in 2:1 ratio) into a spray dryer (180 °C-4 mL/min). A factorial experimental design was used to evaluate the effect of wall material on morphology (circularity and Feret’s diameter) and texture properties of the powders using image analysis. Four formulations using different hydrocolloids were evaluated: (F1) maltodextrin-sodium caseinate (10 wt%), (F2) maltodextrin-soy protein (10 wt%), (F3) maltodextrin-sodium caseinate (15 wt%), and (F4) maltodextrin-soy protein (15 wt%). The moisture, solubility, bulk density, and bioavailability of menthol in the capsules were determined. The results showed that F1 and F2 presented the best combination of powder properties: higher circularity (0.927 ± 0.012, 0.926 ± 0.011), lower moisture (2.69 ± 0.53, 2.71 ± 0.21), adequate solubility (97.73 ± 0.76, 98.01 ± 0.50), and best texture properties. Those suggest the potential of these powders not only as an easy-to-consume and ecofriendly instant aromatic beverage but also as a functional one.

## 1. Introduction

According to their effects on human health—by contact, absorption, or ingestion—plants can be classified as poisonous, narcotic, medicinal, aromatic, and spices. Aromatic herbs represent 0.7% of all medicinal plants. They generate secondary metabolites known as active principles, chemical substances that can perform a harmful or beneficial pharmacological action on a living organism. Thus, their main use is as medicine to treat diseases [1,2,3]. Among all, their use in infusions and tisanes, beverages with medicinal connotations, stands out.

Plant consumption for medicinal purposes is a practice as old as mankind itself. Thanks to herbal preparations of many ancient civilizations such as the Egyptians, Mesopotamians, Greeks, and pre-Columbians, it was possible to build an impressive pharmacopeia that continues to be applied today [2,3,4] with a market amounting to USD 300 billion annually [5,6] due to their multiple health benefits and the global boom of “green consumption” [1].

Among the most consumed aromatic herbs in the worldwide market, chamomile (*Matricaria chamomilla* L.) and peppermint (*Mentha piperita* L.) stand out. Only in 2014, the global production of peppermint was about 92,000 tons [7]. Meanwhile, daily, it is estimated that more than a million cups of chamomile tea are consumed around the world [8]. Peppermint leaves contain tannins, flavonoids, free amino acids, and menthol [9]. Due to its several health benefits, in 2004 it was announced as the “medicinal plant of the year” [10]. According to the Colombian Vademecum of Medicinal Plants (CVMP), its leaf infusion is consumed as a treatment for gastrointestinal conditions such as stomach pain and nausea, and nervous conditions such as nervousness [11]. Several studies have shown its efficacy against several diseases. For example, the administration of capsules of the plant to children with irritable bowel syndrome contributed to a significant decrease in symptoms [12]. Additionally, studies in rats showed that ingestion of the plant generates antidiarrheal activity [13]. On the other side, chamomile is constituted of flavonoids, particularly luteolin, vitamin C, and sesquiterpene lactoses that contribute to its bitter taste [14]. In Europe, it is known as a “cure all” since it is considered to be capable of anything in terms of therapeutic applications [15]. The CVMP mentions its traditional use to treat diarrhea, ulcers, menstrual cramps, and other gastrointestinal disorders. In addition, it is used as an antispasmodic, anti-inflammatory, and bactericide [10]. Studies have shown that its essential oil provides bactericidal action against *Helicobacter pylori* [16] and, its flowers extract, antispasmodic activity (in vitro) [17].

Chamomile consumption, especially as a tea, is generally safe. In 2000, the U.S. Food and Drug Administration (USFDA) authorized its use in dietary supplements and food products. Thus, chamomile and its derivates, such as essential oil, extracts, and distillates, are classified as GRAS (generally regarded as safe) [15]. Although there are no representative health side effects, people with known hypersensitivity to species of Asteraceae/Compositae family, e.g., daisy, marigold, and chrysanthemum, should avoid consuming chamomile to reduce the probability of an allergic reaction [18]. In addition, pregnant women (risk of miscarriage), individuals with asthma (risk of making it worse), diabetics (risk of hypoglycemia or low blood sugar), and hypertensive and hypotensive patients (risk of blood pressure dropping too low) should also avoid chamomile [19]. On the other hand, peppermint should not be consumed by individuals with hypersensitivity to its leaves’ preparations or menthol. Its consumption is not recommended during pregnancy and lactation, nor to patients with gastroesophageal reflux (heartburn may increase) or biliary disorders, and children under 4 years [20].

Now, aromatic herb infusions are part of the tea industry, whose sales, according to Passport^®^ database statistics, have increased over the years and are predicted to continue increasing, especially after the COVID-19 pandemic reinforced preventive healthcare. Thus, in Colombia, from 2019 to 2020, there was a 21% growth where the most sold product was herbal/fruit tea with sales of 22.8 billion USD, a value that is predicted to increase [21]. Despite its health benefits and the great market acceptance of this product, there are two factors against it. First is preparation time. These drinks are usually prepared by soaking leaves/flowers in water for about 5 min [1], plus the time it takes to heat the liquid. Although this time seems short, it is representative if we consider that today everything moves faster, and consumers prefer instant or ready-to-eat products. The second is the packaging. Tea bags are the most common packaging for these beverages. These are usually manufactured from paper with a percentage of food-grade nylon or polyethylene terephthalate (PET) [22]. The high consumption of this product results in a high production of waste that is difficult to degrade due to the presence of plastic materials. This situation contributes in a minor but representative way to global warming by contaminating the surrounding soil and water [23]. However, there is also a health problem because this type of packaging can decompose into micro- and nanoplastics. Since water used for these beverages frequents temperatures of 95 °C or higher, the plastic present in tea bags can degrade, even those of food grade; thus, they release toxic substances when heated above 40 °C. Studies have shown that one plastic tea bag in water heated to 95 °C releases approximately 11.6 billion microplastics and 3.1 billion nanoplastics in a single drinking cup [22].

One way to avoid any side effects that may be caused by microplastic ingestion is to offer aromatic beverages in a presentation that does not include tea bags. In addition, considering the relatively long preparation time of these drinks, an interesting option would be to find a way to produce them in an instant version, i.e., in powder form. Therefore, the aim of this study was to develop a powdered aromatic beverage, starting from a peppermint and chamomile tisane, encapsulating menthol and luteolin using spray drying by testing 3 hydrocolloids as wall materials: maltodextrin, sodium caseinate, and soy protein. The powders obtained were characterized (morphology, texture, moisture, solubility, bulk density, and bioavailability) to have safety approximation (humidity) and to ensure their functionality and quality as an innovative food product for subsequent use as an instant aromatic beverage.

## 2. Materials and Methods

### 2.1. Materials

The peppermint and chamomile were purchased from a local stand located inside the Paloquemao Market Square (Bogotá, Colombia). The wall materials used, such as maltodextrin (95% solids), were obtained from Químicos Mandarín (Bogotá, Colombia), while the sodium caseinate (90 wt% protein) and the soy protein (90 wt% protein) were obtained from CIMPA SAS (Bogotá, Colombia). For the thermogravimetric analysis, pure menthol, more specifically crystal menthol (100% pure), was obtained from MarketQuímicos (Bogotá, Colombia).

The ammonium carbonate ((NH_4_)_2_CO_3_), hydrochloric acid (HCl), pepsin (700 U/g), and pancreatin (50,483 U/g) were purchased from Panreac (ITW Reagents Darmstadt, Germany), sodium chloride (NaCl), and calcium chloride (CaCl_2_) were obtained from Sigma-Aldrich (Merck, St. Louis, MO, USA), and potassium chloride (KCl) from Supelco (Merck, St Louis, MO, USA). The potassium dihydrogen phosphate (KH_2_PO_4_) from Scharlau (Scharlab, Barcelona, Spain), sodium carbonate (NaHCO_3_) from Chemi (Bogotá, Colombia), hydrated magnesium chloride (MgCl_2_(H_2_O)_6_) from J.T Baker (Fisher Scientific Madrid, Spain) and alpha amylase was obtained from ChemCruz (Santa Cruz Biotechnology, Dallas, TX, USA). All were used to model the digestive fluids for in vitro digestion following the methodology described by Minekus et al. [24] with the modifications made by Amaya et al. [25].

### 2.2. Methods

#### 2.2.1. Experimental Design

A factorial design (DOE) 2^2^ (Table 1) with two replicates was used. The response variables to be measured were morphometric parameters of interest (particle size and circularity) and texture homogeneity of the instant powders after the spray drying process. The factors varied were the hydrocolloids as wall material that covers the matrix (maltodextrin + sodium caseinate and maltodextrin + soy protein) and the concentrations of hydrocolloids (sodium caseinate and soy protein, 10 wt% and 15 wt%) in relation to the total wall material. The selection of these hydrocolloids was made on the basis that maltodextrin is one of the most used wall materials for the encapsulation of powders. In addition, supplementary wall materials were chosen to compare the use of an animal protein versus a vegetable protein. All results were subjected to an analysis of variance (ANOVA) performed in Minitab^®^. A Tukey Test with a 95% confidence interval was also performed to compare the difference between means.

#### 2.2.2. Obtaining the Instant Powders

A total of 1.28 kg of peppermint and 1.00 kg of chamomile were used and divided into groups of 171.2 g. Each pile was washed within containers filled with water to eliminate residues that could interfere with the purity, processing, or quantification of the product. Each group was placed on aluminum trays, being careful to not mix them, and then placed in an ultrafreezer (Eppendorf^®^ HEF^®^ U410, Hamburg, Germany) at −80 °C for 7 h. After this time, the trays were placed in a freeze dryer (Labcono ^®^ FreeZone ^®^ 6L, Kansas City, MO, USA) which operates from −40 °C to 15 °C with a ramp of 1.5 °C/min during 70 h and an ultimate pressure of 1.5·10^−3^ mbar.

On a heating plate at 180 °C, an 800 mL beaker covered with aluminum foil was placed with 500 mL of deionized water. This was done to facilitate the water solubility of the compounds of interest (menthol and luteolin). Eight herbal infusions were made by taking 21.4 g of both chamomile and peppermint into the beaker with magnetic agitation at 300 RPM for 10 min. Using a vacuum filtration setup with a Kitasate, each of the prepared infusions were filtered so that the solids interfering with the encapsulation process were removed.

A total of 504 g of maltodextrin, 36 g of sodium caseinate, and 46 g of soy protein were used. These were distributed in each of the filtered infusions in such a way that a 2:1 ratio of wall material versus compounds to be encapsulated was maintained. This process was carried out with constant agitation in a mechanical agitator (Heidolph^®^ Hei-TORQUE^®^ Ultimate 400, Schwabach, Germany) at 300 RPM for 10 min. The formulations used are shown below in Table 2.

Encapsulation sealing was carried out using a mini-spray dryer (BÜCHI^®^ B-290, Flawil, Switzerland) with an inlet air-stream temperature of 180 °C, a feed rate of 4 mL/min-15%, pumping rate, and a suction rate of 90% for F1 and F2 and 70% for F3 and F4 since these formulations had losses due to sticking in the spray dryer bell when using a 90% suction rate. These values were fixed based on a similar I-menthol process [26]. Each sample was fed into the spray dryer using a peristaltic pump as a pumping system. The powder obtained from the collection cup was stored in individual Falcon tubes, covered with aluminum foil, inside Ziploc^®^ bags, and stored in a desiccator until analysis.

#### 2.2.3. Instant Powder Characterization

##### Morphology and Surface Properties

An image analysis was performed to evaluate the morphology and texture homogeneity of the powders in ImageJ^®^. Based on the methodology of Eratt et al. [27] with some modification, a scanning electron microscope (SEM) was used (Thermo Scientific^TM^ Phenom Pro X G6 Desktop SEM, Waltham, MA, USA) with a voltage of 15 kV. It was necessary to metalize the samples previously by using a metalizer (Denton Vacuum^®^ Desk V TSC, Moorestown, NJ, USA). This process was carried out with gold and UAP argon gas at 20 mA for 1 min.

For morphology, images were obtained at a magnification of 510×, where 2 parameters of interest (circularity and Feret’s diameter) were calculated for 10 capsules per formulation using a conversion factor of 3927 pixels/micra. On the other hand, to determine homogeneity, images were obtained at a magnification of 3600×. For each formulation, 6 crops were measured with a size of 350 × 350 pixels; additionally, a surface plot and the values for 6 parameters were obtained: fractal dimension texture (FDt), angular second moment (ASM), contrast, correlation, inverse difference moment (IDM), and entropy.

##### Moisture

The methodology mentioned in the book on the determination of moisture content was used [28], with some variation. The value of this property was obtained by using a thermobalance (Precisa ^®^ XM 60, Dietikon, Switzerland). For each formulation, 3 g of instant powder were used.

##### Bulk Density

This parameter was calculated considering the definitions of Abdullah and Geldart [29] and Artamonov et al. [30]. Independently for all formulations, 2 g of powder were weighed on an analytical balance (Vibra-HTR ^®^, Tokyo, Japan) and introduced into a 10 mL test tube with the aid of a paper funnel. Subsequently, 10 dry hits were given on a flat surface and the volume value indicated on the test tube was taken.

##### Solubility

This property was calculated as a combination of the methodologies proposed by Serna-Cock [31] and Largo-Avila [32]. For each formulation, 1 g of instant powder was solubilized in 50 mL of distilled water. This mixture was stirred on a plate with magnetic stirring at 1150 RPM for 5 min at 25 °C. The solution was placed in a Falcon-type tube and centrifuged at 3000 RPM for 5 min at 25 °C. An aliquot of 25 mL of the supernatant was taken and transferred to a Petri dish that was placed in an oven at 105 °C for 5 h. After this time, the solution was weighed again. Powder’s solubility percentage was calculated by the weight difference of the supernatant aliquot, as shown below (1)

(1) $$Solubility\;in\;water=\frac{Initial\;weight\;of\;the\;aliquot\;\left(g\right)-Final\;weight\;of\;the\;aliquot\left(g\right)}{Initial\;weight\;of\;the\;aliquot\left(g\right)}$$

##### Controlled Release of the Powder Contents

An in vitro digestion test was performed to corroborate whether the capsules can protect the menthol and luteolin until they reach the small intestine, where they are expected to be released. This procedure was carried out only for the two formulations that presented the best results for morphology, texture, moisture, and solubility. For this, salivary, gastric, and intestinal environments were simulated. The procedure was performed using the methodology of Minekus et al. [24] with modifications made by Amaya et al. [25]. For each of the 3 environments (SSF, SGF, and SIF), 50 mL of simulated fluid were prepared using an Erlenmeyer flask capped with aluminum.

Simulated Salivary Fluid (SSF)

Simulated salivary fluid was prepared with 1.3% (*v*/*v*) α-amylase (5680 U/mL), 85.5% (*v*/*v*) salivary base fluid, 0.2% (*v*/*v*) deionized water and 10% (*v*/*v*) menthol and luteolin capsules. The pH was adjusted to 7 with the addition of 0.1 M NaOH solution and the temperature was kept at 37 °C. This stage lasted 2 h with constant agitation at 100 RPM.

Simulated Gastric Fluid (SGF)

To prepare this fluid, 1.0% (*v*/*v*) pepsin (700 U/g), 88.27% (*v*/*v*) gastric base fluid, 0.03% (*v*/*v*) 0.3 M CaCl_2_, and 0.7% (*v*/*v*) deionized water were added. The remaining 10% (*v*/*v*) corresponded to the powder. The pH was adjusted to 3 with the addition of a 0.1 M HCl solution and the temperature was kept at 37 °C. This stage lasted 2 h with constant agitation at 100 RPM.

Simulated Intestinal Fluid (SIF)

This fluid was prepared with 0.2% (*v*/*v*) pancreatin (50,482 U/g), 89.0% intestinal base fluid, 0.1% (*v*/*v*) 0.3 M CaCl_2_, and 0.7 (*v*/*v*) deionized water. The remaining 10% (*v*/*v*) corresponds to the powder. The pH was set to 7 with the addition of 0.1 M NaOH solution and the temperature was maintained at 37 °C with constant agitation at 100 RPM. This phase had a duration of 3 h.

##### Concentration of Menthol Released

First, a UV-Vis spectrophotometer (Thermo Scientific^TM^ GENESYSTM 10S, Madison, WI, USA) was used to evaluate the wavelength at which menthol absorbs the most photons (Supplementary Materials). Menthol acts as a compound that does not absorb UV rays due to its lack of chromophore groups. For this, it must undergo a chemical derivatization process to be replaced by a UV-sensitive chromophore. For this reaction, a dye reagent is used and, due to the low reactivity of menthol, this must occur in a strongly acidic medium [33,34]. In this case, one of the two methodologies proposed by [35] was used. Here, vanillin is used as a coloring reagent and concentrated sulfuric acid as a medium supplier, resulting in a stable purple product.

The menthol sample used for length sweep was prepared in a 5 mL test tube to which were added 2.5 mL of concentrated sulfuric acid, 50 mg of pure menthol, and 0.5 mL of a 1% (*w*/*v*) solution of vanillin that was also prepared with concentrated sulfuric acid. Everything was then diluted with deionized water to the volumetric mark.

To find the concentration of menthol released into the intestinal environment, the sample was prepared based on pure menthol. In a 5 mL tube test, 2.5 mL of sulfuric acid, and 0.5 mL of 1% (*w*/*v*) vanillin solution were added and the sample was volumetrically diluted with the simulated intestinal fluid (2 mL). In this case, deionized water was not used because it was already contained in the SIF. To prepare the blank, approximate amounts of wall materials within the powders were considered based on TGA results (data not shown). The concentration was calculated using the Beer–Lambert law (2) using a quartz cell pitch length (*b*) of 1 cm and a molar extinction coefficient for menthol (*ε*) of 1.82386 × 10^4^ M^−1^cm^−1^. The percentage of menthol released in the SIF was calculated using (3).

(2) A=ε×b×C

(3) $$Menthol\;released\left(\%\right)=\frac{amount\;of\;menthol\;released(g)}{amount\;of\;menthol\;encapsulated(g)}\times 100$$

## 3. Results and Discussion

### 3.1. Morphology

**Figure 1:**
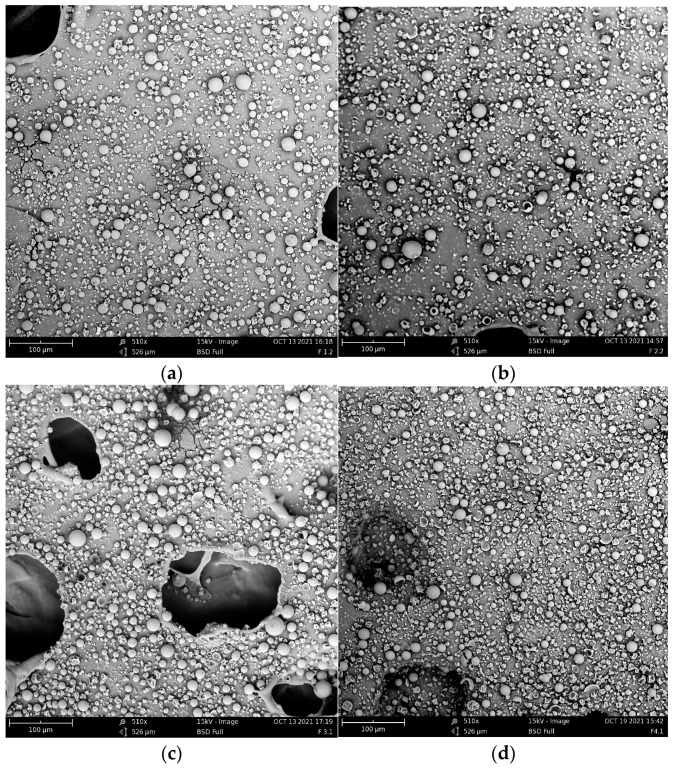
SEM images for morphological analysis of each formulation: (**a**) F1; (**b**) F2; (**c**) F3; (**d**) F4.

Figure 1 shows images of the four powder formulations that were used for the measurement of the two morphological parameters of interest. Powders do not appear to exhibit high agglomeration and capsules are seen to be mostly almost spherical. When compared to similar works on the elaboration of instant powders reported for *Hibiscus sabdariffa* L. [36] and in the encapsulation of bioactive compounds [37], this type of behavior and the geometry of the particles were also observed, so the fact that no cracks or holes were observed in them may be an indication that the compounds of interest were adequately coated. In addition, F1 appears to have lower particle sizes than the other formulations, while F3 is the largest one.

Table 3 presents the values of circularity and Feret’s diameter obtained for each formulation through ImageJ software analysis. Circularity values between formulations F1 and F2 were not statistically different (*p* > 0.05), as well as between F3 and F4. However, there is a meaningful difference between formulations with 10 wt% and 15 wt% concentrations. In general, the first ones obtained the highest value of this property. As seen in similar studies, when using maltodextrin as the main wall material, in conjunction with sodium caseinate and soy protein, the particle shape tends to be spherical and smooth [38,39].

For powder food, one of the objectives is to ensure that the powders have good properties to facilitate their rehydration. To achieve this, powders must present high agglomeration since this phenomenon improves functionalities such as dispersion, solubility, and wetting properties [40]. If an instant product is to be achieved, powders require agglomeration to improve their reconstitution [41,42]. In a general way, spray drying methodology is used to produce agglomerated powders, but a variable that greatly influences achieving this state is the particle size. Large particle sizes are desirable to achieve good agglomeration, especially to increase dispersibility, which has been found to decrease with high percentages of fine particles (below 90 μm) [40,43,44,45,46]. Small particle sizes with symmetrical shapes are disposed to form compact particle packings that inhibit water penetration. In contrast, larger particle sizes provide greater space between interstices for wetting [47]. The results of this parameter are also shown in Table 3, where F3 and F4 are the best formulations since they have the highest values for this property. For this attribute, they did not present a statistically important difference, the same situation with F4-F2 and F1-F2. Meanwhile, F3 and F2-F1 did present considerable variance.

The later results obtained for morphometric parameters can be explained because, at the time of preparing the infusions, it was observed that the formulations with 15 wt% of supplementary wall material (F3 and F4) presented a greater amount of agglomeration during the mixing of the solution and the wall material. This was very unstable and not quite homogeneous so that, in the beginning, one could have a homogeneous solution with a single phase, but after some time, compounds of both caseinate and soy protein precipitated. Thus, this situation produced larger particle sizes with relatively low circularity. If the feeding is not homogeneous, it can result in several droplet size distributions. In such cases, the feeding not only lacks homogeneity but also becomes thicker compared to the feeding solutions for F1 and F2. As a result, it mainly consists of large droplet sizes, leading to the formation of larger, wrinkled, and less circular powder particles [48]. This can be observed in Table 3.

### 3.2. Surface Properties

**Figure 2:**
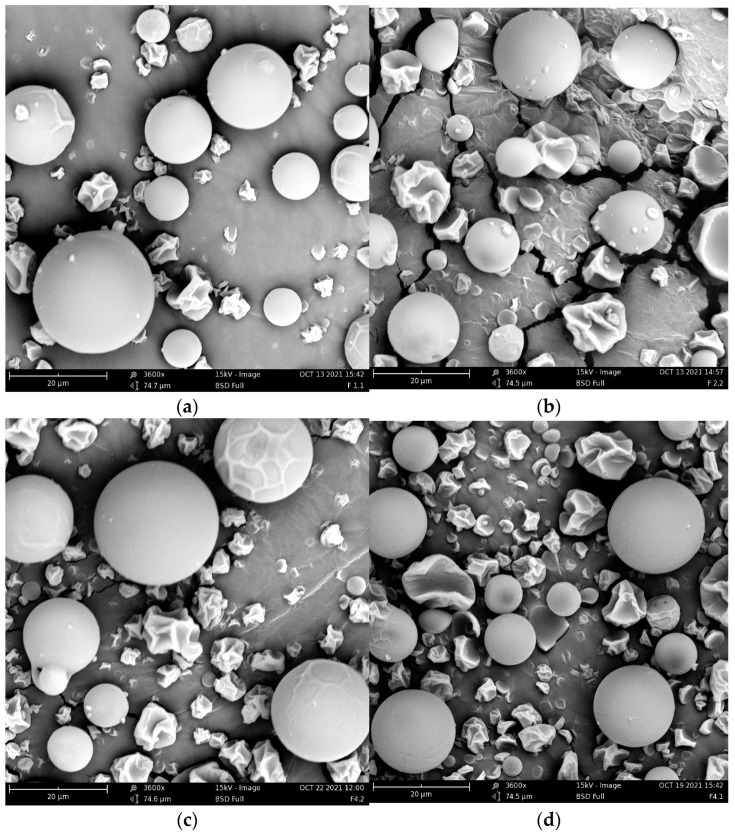
SEM images for image analysis of each formulation: (**a**) F1; (**b**) F2; (**c**) F3; (**d**) F4.

Figure 2 contains some of the images used to analyze the surface parameters of interest. It can be observed that for all four formulations, the most common shape was a smooth-surfaced sphere of different sizes. According to the work of Sambroska et al. [39], the morphology of the particles is a good indicator for knowing their nature, whereas a spherical and smooth surface geometry indicates that these are amorphous. Images also show hollow or wrinkled particles. This phenomenon may have been caused by the operating conditions of the spray dryer. Hollow capsules are more fragile and can fragment when they collide with others. This type of particle can affect powder properties such as bulk density. According to Both et al. [48], powders containing a higher content of hollow particles have lower bulk densities. In summary, it can be said that all the formulations presented an amorphous nature and good structural definition. On the other hand, for F2 and F4, the presence of dents can be noted due to caseinate shrinkage and possible uneven drying of the powders [44]. The addition of these surface properties is a novel contribution to this work since this type of analysis is rarely in other studies.

**Figure 3:**
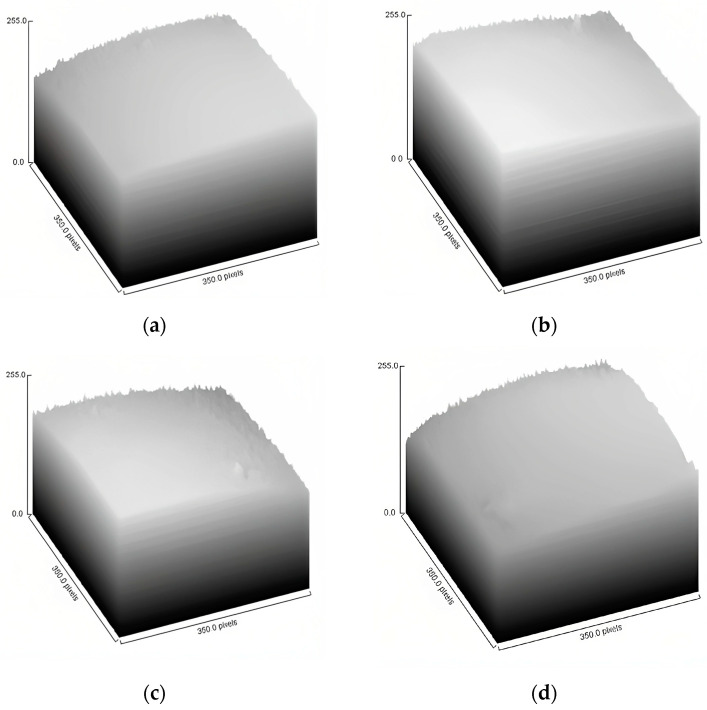
Surface plots for texture analysis of each formulation: (**a**) F1; (**b**) F2; (**c**) F3; (**d**) F4.

Surface plot graphs are presented in Figure 3. This type of plot is used to analyze the heterogeneity of the samples at a given crop size. At first glance, it can be seen how the formulations made with a concentration of 10 wt% (F1 and F2) presented a more homogeneous and smoother surface compared to those made with 15 wt% (F3 and F4). Similarly, powders that were prepared with soy protein have a more heterogeneous and irregular surface as they present more peaks.

Table 4 shows the values of the texture parameters obtained. Analyzing FDt, the ideal is to have a low value since a high value indicates rougher and irregular surfaces [49]. Thus, although there were no statistically significant differences in this parameter among the formulations, F4 was the one with the lowest value. Similar studies showed that this factor is relevant for evaluating the effects of the different treatments [50]; however, in this study, since no statistically significant differences were observed, it can be said that this is not a relevant parameter for quantifying or evaluating the effect of the different factors (material and concentration of the supplementary wall material) on the surface properties. In the case of ASM, higher values increase the uniformity of the samples. When performing the Tukey Test, between F1 and F2 and among F1, F3, and F4 there were no statistically significant disparities, and the first two were those with the highest values. Meanwhile, among F2 and F3-F4 there was a relevant difference. This property is like the inverse different moment (IDM) with the difference that higher ASM values indicate higher directional uniformity [51]. The difference between F2 with F3 may be given by the selection of the supplementary wall material while the difference between F2 and F4 may be given by the concentration of the supplementary wall material. Despite this difference, it has been evidenced in several studies that this property is not significant [49].

In terms of contrast, this one should ideally have low values because it represents the local variation in the contrast between a pixel and the next one. Tukey’s test showed that there was no important variation among F1, F2, and F4, as well as between F2 and F3. However, between F1 and F3 a difference was present and the last one was the formulation with the lowest value. Higher values indicate a rougher and more heterogeneous surface [49]. In this study, the difference between F1 and F3 could be attributed to the concentration of the supplementary wall material for sodium caseinate. Although there is a significant difference between F1 and F3, other authors mention [49,50,51,52] that this property is not significant for estimating product quality. On the other hand, it is desirable to have correlation parameters that are high values since this is the measure of how correlated a pixel and its neighbor are. When performing Tukey’s Test, there was no statistically significant difference among all formulations. This shows that this parameter is not optimal for evaluating the effect of the supplementary wall material or concentration on the image. In addition to the contrast, other authors agree that this property is not significant for estimating product quality [49,50,51,52].

Finally, it is desirable for IDM results to be high since it indicates the homogeneity of the image. The higher the value, the greater the homogeneity. It was observed that F1, F2, and F3 were not statistically significant differences among themselves, as well as formulations F1, F3, and F4. Meanwhile, F2 and F4 did present meaningful variances. According to Laddi et al. [52] this parameter has a strong correlation with high-quality product samples, since it is related to uniformity in size and is connected to the spatial arrangement of powder particles, and is closely related to their shape. A higher level of homogeneity indicates that the powder particles are consistent in size and shape, which is considered a desirable trait in the evaluation of product quality. In this case, since there is a significant difference between F2 and F4, it can be concluded that the concentration of the supplementary wall material of soy protein is a significant factor to obtain a more homogeneous image.

On the other hand, when analyzing entropy, it is sought to have low values because it is the measure of image randomness. The results showed that there were no important differences among formulations F1, F3, and F4 and among F1, F2, and F4, while between F2 and F3 there was a statistically significant difference. This difference between the formulations could be related to the difference in the homogeneity of their structures since higher values represent a more heterogeneous particle [51]. This parameter has been found to be useful to evaluate changes in the microstructure of the particles, as Barrera et al. [50] make use of it to evaluate the consequences of mechanical damage in the particles and evidence a significant difference between damaged and normal particles. Similarly, Laddi et al. [52] show how entropy values show degradation in tea quality; therefore, high values are not desired. However, Hernandez-Carrion et al. [49] mentioned not finding a predictable trend for this value, since, in some cases, increasing the magnification significantly reduced this property, while in others it did not produce significant changes. Nonetheless, it has been shown that this parameter is useful to describe microstructural changes. The lowest results were obtained in F2, possibly due to the good homogeneity of its structure, as shown in the IDM.

### 3.3. Moisture

This parameter is often used as an index of product stability; therefore, the moisture of foods is of great interest. Moisture is understood as the amount of water present in a medium that can influence the preservation or resistance to spoilage of products [53]. For granulated foods, a high percentage of moisture indicates that cohesion forces between particles are greater, a phenomenon that can affect their dispersion speed [54].

The values of this property for each formulation are shown in Table 5. It can be evidenced that all four formulations did not present statistically significant differences among them. Using hydrocolloids such as maltodextrin as part of the wall material can lead to higher moisture values because it may have high dextrose content. Maltodextrins with high dextrose content present a higher number of branches with hydrophilic groups and, therefore, water molecules from the environment can adhere more easily to the powders [55]. Even so, the formulations evaluated presented low moisture values, a fact that contributes to good product stability. If milk powder is taken as a reference, it is known that for this, food moisture percentage has an upper limit of 4% [41]. Similar studies on sugarcane-juice powders also show the importance of having low values of this property, since it directly affects the shelf life of the powders. It was reported that the moisture content of these powders was between 3.02 and 3.24%, which shows a resemblance with similar commercial spray powders [56]. When comparing this percentage with the obtained in the powders studied, it is observed that these are within the common range for this type of product.

### 3.4. Bulk Density

It is important to emphasize that this is not an intrinsic property of the powder since it can vary according to its handling [57]. This parameter can be affected by many factors, one of these being the drying method and drying conditions (temperature, time, and pump power). These not only have an impact on the apparent volume but also on the shape of the particles [58]. Now, Table 5 also shows the results for each formulation. It was observed that formulations containing caseinate had a lower density than those containing soy protein. This is because bulk density is inversely proportional to particle size, i.e., the smaller the particle, the higher the bulk density [39]. Similarly, Bai et al. [59] showed that microcapsules with spherical shapes and regular surfaces could be packed more tightly, which results in higher values of bulk density. This can be seen in F3 since it had the largest particle size and the lowest circularity value, making it have the lowest bulk density value. This property is useful for calculating other parameters such as porosity and thus to know the fluidity of the powders; it is also an important parameter to consider before packaging the powder since it indicates their cohesiveness. If the product is very cohesive, it will not be easy to disperse in the medium, a situation that would affect the dissolution rate. For this reason, very small particle sizes are not sought to avoid very cohesive powders. Low bulk density of the powders is desired due to the “puffing” effect to increase the sensory acceptance of the consumers [58]. If the material is porous, it will have greater contact with the medium, thus facilitating the dispersion of the powders in the solution [55].

### 3.5. Solubility

Serna-Cock et al. [31] define solubility as the rate at which compounds present in powder particles dissolve in water. This is a factor of great importance when presenting a product for reconstitution on the market. Depending on how easily a powder can be dispersed in the liquid phase, the consumer’s decision to purchase it can be influenced. Since the objective of this property is to present values close to 100%, wall materials with high solubility percentages are recommended for use.

Table 5 shows the solubility percentages obtained for each formulated powder. It was observed that among formulations F1, F2, and F3 there was no statistically significant difference for this attribute, as well as among F2, F3, and F4. However, between F1 and F3, there was a meaningful change in their means. F4 showed the highest value of solubility, while F1 had the lowest value. In general, formulations elaborated with soy protein as the supplementary wall material were more soluble than those containing caseinate. Furthermore, higher values of this property were obtained with a concentration of 15 wt%. The solubility of powders depends on several factors: microstructure, density, particle size, spray dryer operating conditions, humidity, and, as already mentioned, the selection of a highly soluble wall material such as maltodextrin, sodium caseinate, or soy protein and their concentration. Similar studies also showed that the shape of the particles affects solubility, since round and rough surface particles have higher values of this property, while elongated and smooth particles are more hydrophobic [57]. For this reason, when powders were placed in water, they were easily reconstituted; however, it was not a homogeneous mixture and after some time the powder precipitated again. Generally, a powder with low hygroscopicity, low water content, and high solubility is considered a good powder [60]. Furthermore, when compared with similar studies of instant powders based on red dragon fruit peel extracts [61] and *Hibiscus sabdariffa* L. (soluble roselle) [36], it can be confirmed that the results obtained were good since the solubility values presented in the other studies ranged between 92.86–97.13% and 90.22–96.86% respectively. Similarly, in the study by Cam et al. [62], a solubility of peppermint extract powder of 97% was observed which was described as optimal for incorporation into food products. Based on the latter characteristic, it could be considered that the product obtained could be attractive for the market.

### 3.6. Controlled Release of the Compounds of Interest (Menthol)

Evaluating the results obtained for the different evaluated parameters, and considering the interest in low moisture, high solubility, high morphologic values, and the best textural characteristics, it was concluded that the two best formulations were F1 and F2. Therefore, an in vitro digestion study was carried out for these two only.

Wavelength sweeps were performed for simulated intestinal fluids and menthol. It was observed that the wavelength where the maximum absorption (λ_max_) occurs agrees with the one reported by [35], which is 525 nm (Supplementary Materials). Regarding the scans presented by the SIF of F1 and the SIF of F2, a wavelength value was obtained where the maximum absorption is displaced from 525 nm, and it was the same for both formulations (461 nm). This result does not imply that the same substance is not being detected, but rather that there are many interferents. The first, and perhaps most important, is luteolin. Since this pure compound was not available, its amount could not be considered when preparing the blank to quantify the menthol. Secondly, there are various compounds of peppermint and chamomile that could have been extracted during the infusion and encapsulated together with menthol and luteolin that were not considered at any time in this work. Additionally, it should be kept in mind that the reference being used is pure menthol; however, it is unlikely that the menthol that was successfully extracted from peppermint was free menthol (I-menthol). Although this compound is mostly found in this plant, it is a slightly water-soluble substance, so it is more likely that menthol was extracted in combination with other compounds [14]. Even so, the presence of menthol in the powders is undeniable, mainly for two reasons. The most obvious is the smell that the powders emanated when they were dissolved in water. The second reason is that the samples prepared with the simulated intestinal fluids showed the coloration produced by the menthol derivative. As already mentioned, this compound gives a purple color. This color, when combined with the brown tone produced by the powders in the SIF, was expected to produce a final dark coloration in the sample, tending to be black, and this is what was obtained. Thus, for all the reasons already mentioned, the absorbance value used in the Beer–Lambert law was the one obtained at the λ_max_ of the reference (525 nm)

Now, analyzing the in vitro digestion results, molar concentrations in the SIF of 6.00 × 10^−6^ M for F1 and 6.15 × 10^−6^ M for F2 were obtained. Although the molar concentration of menthol in the SIF for F2 is slightly higher than the one obtained for F1, the amount of initial menthol that entered the digestion process must be considered. Therefore, the important value is the percentage of menthol released into the intestinal environment. For this parameter, F1 was the best formulation as it allowed the highest amount of menthol to be released into the simulated intestinal fluid (9.36%) while the value for F2 was 4.37%.

Overall, the percentages of menthol released into the SIF for the two formulations were low. This can be seen since, in similar works, the values obtained for the compounds released in this stage of simulated fluid are higher. For instance, in the work of Dumitrascu et al. [63], the release of bioactive compounds from cornelian cherry fruit was studied in which a release of 75% was shown. In addition, using a hydrocolloid mixture of maltodextrin and soy protein results in a higher release of the compound of interest than using soy protein as the only wall material (58%). Similarly, in the work of Amaya Cano et al., [25] an approximate release of 42.2% was obtained in the SIF. The low release in the previous phases is attributed to the low catalytic effect between alpha amylase and sodium caseinate for the SSF and the isoelectric point of caseinate for the gastric phase since it allows a greater attraction between the wall material and the encapsulated oil to improve the protection of the components [25]. As mentioned by Dumitrascu, higher release values are obtained by combining both hydrocolloids (maltodextrin and sodium caseinate) than by using only maltodextrin. The low results can be attributed to the possibility that much of the menthol was released in the previous digestive stages. Since maltodextrin is the majority wall material in both formulations, it is more likely that the release occurred to a greater extent in the simulated salivary fluid due to the interaction of the alpha bonds of maltodextrin with α-amylase. Keeping in mind that F1 and F2 possessed the same amounts of maltodextrin, and both possess a supplementary wall material concentration of 10 wt%, their only difference is the supplementary wall material used. Therefore, their behavior during the gastric phase could have been the key factor for F1 to present a better percentage of menthol released in the SIF. Thus, it is possible that the sodium caseinate present in this formulation was able to retain the menthol in the SGF to a greater extent than the soy protein. The reason for this would be the low pH in this environment which, being below the isoelectric point of the caseinate, produced the denaturation of the caseinate making it insoluble [64,65].

## 4. Conclusions

It was observed that all four powder formulations obtained considerably high and promising solubility values when reconstituting these capsules in water. In addition, capsules presented other good properties of interest such as low moisture, which is ideal for instant products since they present low probabilities of growth of microorganisms. It was seen that morphology affected other properties, such as density since the powders with larger particle sizes had lower density. In addition, consumer interest in this product would be boosted since there is a high frequency of consumption of herbal infusions, as observed in the Passport^®^ report presented in Section 1. The results suggest a high potential in the formulated powders at the time of developing a new product that is innovative, easy to consume, environmentally friendly, and can be marketed as an instant aromatic beverage in the market. For future studies, it is recommended to evaluate in vitro digestion of luteolin also, not only on SIF but also on SGF and SSF. It would be important to study other aspects of these beverages such as stability, rheology, and sensory properties.

## Figures and Tables

**Table 1:** Experimental design.

Factor 1: Maltodextrin Supplementary Wall Material	Factor 2: Concentration of Maltodextrin Supplementary Wall Material	
	10 wt%	15 wt%
Sodium caseinate	F1	F2
Soy protein	F3	F4

**Table 2:** Formulations used to obtain the mixture used to feed the spray dryer.

Compound	F1	F2	F3	F4
Maltodextrin	15.41 ^1^	15.41	14.55	14.55
Sodium Caseinate	1.71	0	2.57	0
Soy Protein	0	1.71	0	2.57
Total dissolved solids (wall material)	17.12	17.12	17.12	17.12
Water	74.32	74.32	74.32	74.32
Peppermint	4.28	4.28	4.28	4.28
Chamomile	4.28	4.28	4.28	4.28
Total aqueous phase	82.88	82.88	82.88	82.88
Total mix	100	100	100	100

^1^ Values presented in wt%.

**Table 3:** Results for morphometric parameters.

Formulation	Wall Material	wt%	Circularity	Feret’s Diameter (μm)
F1	MD + CS	10	0.927 ^a 2^ (0.012) ^1^	13.87 ^c^ (0.68)
F2	MD + PS	10	0.926 ^a^ (0.011)	15.89 ^b,c^ (0.79)
F3	MD + CS	15	0.878 ^b^ (0.024)	19.23 ^a^ (2.003)
F4	MD + PS	15	0.895 ^b^ (0.012)	17.88 ^a,b^ (1.16)

^1^ Values in parenthesis represent the standard deviation. ^2^ For the same column, different letters indicate the existence of statistically significant differences according to the Tukey Test (*p* > 0.05).

**Table 4:** Results for texture parameters where IDM stands for inverse difference moment, FDt stands for fractal dimension texture, and ASM stands for angular second moment.

Formulation	Wall Material	wt%	Entropy	IDM	FDt	Correlation	Contrast	ASM
F1	MD + CS	10	7.60 ^a,b 1^ (0.35) ^2^	0.156 ^a,b^ (0.022)	2.52 ^a^ (0.03)	2.00·10^−3 a^ (9.4·10^−4^)	182.385 ^a^ (18.78)	6.38·10^−4 a,b^ (2.05·10^−4^)
F2	MD + PS	10	7.33 ^b^ (0.41)	0.172 ^a^ (0.027)	2.53 ^a^ (0.14)	2.33·10^−3 a^ (8.6·10^−4^)	158.923 ^a,b^ (30.69)	8.51·10^−4 a^ (2.49·10^−4^)
F3	MD + CS	15	7.87 ^a^ (0.28)	0.151 ^a,b^ (0.031)	2.50 ^a^ (0.06)	1.67·10^−3 a^ (5.4·10^−4^)	136.645 ^b^ (33.26)	5.21·10^−4 b^ (1.55·10^−4^)
F4	MD + PS	15	7.84 ^a,b^ (0.26)	0.133 ^b^ (0.015)	2.50 ^a^ (0.02)	1.99·10^−3 a^ (6.8·10^−4^)	193.138 ^a^ (13.25)	5.44·10^−4 b^ (1.68·10^−4^)

^1^ For the same column, different letters indicate the existence of statistically significant differences according to the Tukey Test (*p* > 0.05). ^2^ Values in parenthesis represent the standard deviation.

**Table 5:** Results for properties of interest of the powders.

Formulation	Wall Material	wt%	Solubility (%)	Moisture (%)	Bulk Density (g/mL)
F1	MD + CS	10	97.73 ^b 2^ (0.76) ^1^	2.69 ^a^ (0.53)	0.250 ^b^ (0.020)
F2	MD + PS	10	98.01 ^a,b^ (0.50)	2.71 ^a^ (0.21)	0.302 ^a^ (0.046)
F3	MD + CS	15	97.99 ^a,b^ (0.20)	3.11 ^a^ (1.41)	0.248 ^b^ (0.07)
F4	MD + PS	15	98.29 ^b^ (0.29)	2.78 ^a^ (1.28)	0.291 ^a,b^ (0.038)

^1^ Values in parenthesis represent the standard deviation. ^2^ For the same column, different letters indicate the existence of statistically significant differences according to the Tukey Test (*p* > 0.05).

## Data Availability

The data presented in this study are available on request from the corresponding author. The data are not publicly available by decisions of the authors.

## Supplementary Materials

The following supporting information can be downloaded at: https://www.mdpi.com/article/10.3390/foods12102080/s1.
