# Supplementary figures and images for: Encapsulation of Menthol and Luteolin Using Hydrocolloids as Wall Material to Formulate Instant Aromatic Beverages
Source: Foods. 2023 May 22;12(10):2080. doi: 10.3390/foods12102080 (PMC10217312; doi:10.3390/foods12102080)

# Absorption Spectrum

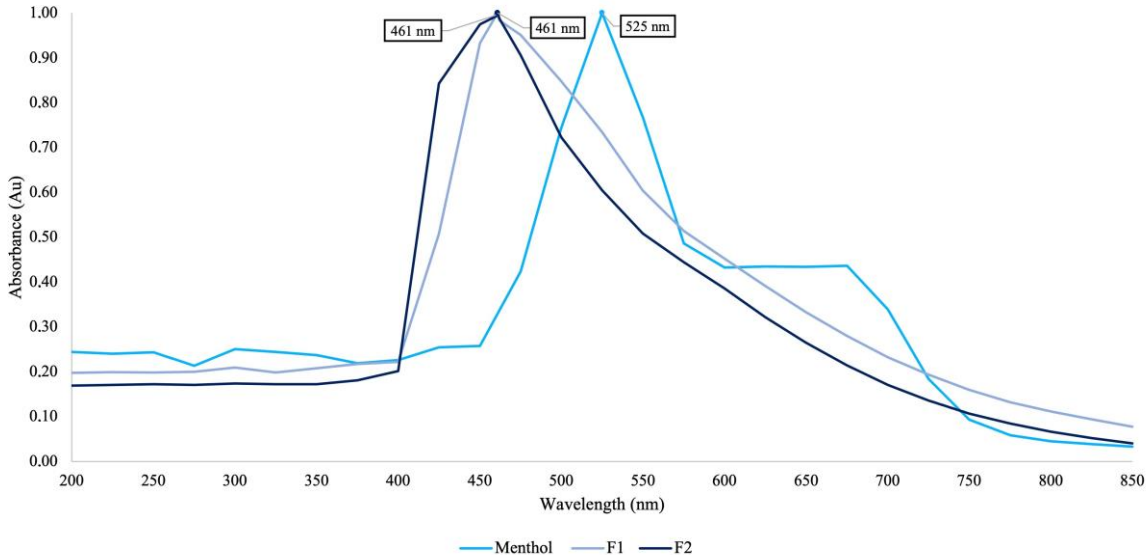

Supplement: Supplementary file 1 [file foods-12-02080-s001.zip › foods-2398783-supplementary.pdf]
